# The Severity of Hepatitis D in Young Adults of Age 18-25 Years

**DOI:** 10.7759/cureus.10855

**Published:** 2020-10-08

**Authors:** Zaigham Abbas, Muhammad Ali Qadeer, Haider A Mandviwalla, Minaam Abbas

**Affiliations:** 1 Gastroenterology and Hepatology, Dr. Ziauddin University Hospital Clifton, Karachi, PAK; 2 Gastroenterology and Hepatology, School of Clinical Medicine, University of Cambridge, Cambridge, GBR

**Keywords:** hepatitis delta, hepatitis b, cirrhosis, young adults, statistical model, child class, meld score

## Abstract

Background

Current literature on the prevalence and characteristics of hepatitis D virus (HDV) infection in young adults is limited. This study aims to determine the disease characteristics and severity in young adults.

Methods

The case records of HDV RNA positive patients of age 18-25 years were analyzed.

Results

Out of 119 patients, 105 (88%) patients were male. HBV-DNA was detectable in 83 (70%). Hepatitis B e-antigen (HBeAg) was non-reactive in 99 (83%). Cirrhosis was identified in 45 (37.8%) individuals; nine (7.5%) were classified as Child class B or Child class C. Twenty-four (20.2%) had a Model For End-Stage Liver Disease (MELD) score of ≥10, out of these 16 had a score of 15 or more. The risk of decompensation was calculated according to the Baseline-event-anticipation (BEA) score; eight (6.7%) patients were at BEA-A (mild risk), 105 (88.2%) were at BEA-B (moderate risk), and six (5.0%) were at BEA-C (severe risk). Notable findings in patients with cirrhosis included splenomegaly, low total leucocyte counts, low platelets, high bilirubin, elevated aspartate aminotransferase, gamma-glutamyl transferase and international normalization ratio, low albumin, high AST to Platelet Ratio Index (APRI), and high BEA score. The splenic size, platelet count, and albumin levels were independently associated with cirrhosis (p < 0.001, <0.001, and 0.003). A model using a combination of platelet count, albumin, and spleen size was developed to accurately predict cirrhosis in this cohort. It had an area under the receiver operating characteristics (AUROC) of 0.935.

Conclusions

HDV-infected young adults, age 18-25 years, were at moderate to severe risk of disease progression. About one-third of patients had already developed cirrhosis indicating the aggressive nature of the disease.

## Introduction

Approximately 12 million persons are infected with hepatitis D worldwide [1]. The incidence of hepatitis D virus (HDV) remains high in Asia and is a significant cause of morbidity [2]. In adults, co-infection of hepatitis B virus (HBV) with HDV is usually self-limiting, but can also be associated with a severe form of acute hepatitis and may lead to fulminant hepatitis [3]. Approximately 2% of patients with co-infection progress to cirrhosis. On the other hand, the superinfection of HDV on HBV eventually leads to chronic HDV infection in 90% of all cases. This, in turn, results in cirrhosis in 70% of patients within five to 10 years of the acquisition of the superinfection [4]. The progression to cirrhosis is more rapid, and the incidence of cirrhosis is three times higher with HDV superinfection when compared with HBV mono-infection [5]. Patients with chronic HDV have higher transaminase levels, lower platelet counts and prothrombin activity, and a higher incidence of necroinflammation and significant fibrosis on liver biopsy [6].

HDV infection is an important cause of progressive liver disease in children who are chronic hepatitis B surface antigen (HBsAg) carriers [7]. The acquisition of HDV infection occurs primarily through horizontal transmission [8]. Children infected with HDV typically have minimal symptoms and high levels of HBV replication in the liver with the presence of hepatitis B core antigen (HBcAg) in the liver and hepatitis B e-antigen (HBeAg) and HBV DNA in the serum [9]. We previously compared 48 children suffering from hepatitis D with 48 children with hepatitis B monoinfection. Cirrhosis was seen in 13 (27%) cases with hepatitis D compared with two (4%) with hepatitis B monoinfection. Six patients with hepatitis D had decompensation at the time of presentation, whereas only one patient with HBV monoinfection had decompensation [10].

We, therefore, postulated that if children with HDV infection have a more aggressive form of liver disease than those with HBV monoinfection, patients with HDV in their young adulthood would also display an accelerated course of progression of the disease, with a greater risk of having cirrhosis and decompensation. Existing literature on the prevalence and characteristics of HDV infection in the young adult population between 18-25 years is limited. This study aims to ascertain disease characteristics and severity of hepatitis D in the young adult population.

## Materials and methods

A retrospective study of records of HDV RNA positive patients, aged between 18-25 years, who visited our liver clinic, was conducted. Patients with an incomplete profile were excluded. A diagnosis of cirrhosis was made based on ultrasonographic findings. Cirrhosis was considered decompensated in patients that had variceal bleeding, ascites, or hepatic encephalopathy. The risk of decompensation was categorized with the help of baseline-event-anticipation (BEA) Classification [11]. The scoring system is based on male gender = 1, age > 40 = 1, Eastern Mediterranean origin = 1, platelet count <100 = 1, platelet count <50 = 2, and bilirubin > upper limit of normal = 1. BEA-A = 0-1 point, BEA-B = 2-4 points, BEA-C = 5-7 points. The Ethics Review Committee reviewed the study protocol and granted access to the data. Our study did not require written/informed consent because of retrospective analysis without disclosing the identity or doing any intervention. The study protocol conforms to the ethical guidelines of the 1975 Declaration of Helsinki as reflected in a priori approval by the institution's human research committee.

All analyses were conducted using the SPSS software (IBM SPSS Statistics Base 26, IBM, Armonk, New York, USA). Categorical variables were compared by Chi-square or Fisher’s exact test, as appropriate. For all continuous variables, the mean, standard deviation, median, and range were noted. For comparisons between groups, a non-parametric independent sample Mann-Whitney U test was used, with the significant alpha level (p-value) set at 0.05. Linear regression analysis was conducted to identify independent factors associated with cirrhosis and receiver operating characteristic (ROC) curves were drawn for these factors. Binomial logistical regression was then used to produce a predictive model combining the three factors and ROC curves for these models were produced.

## Results

A total of 119 patients were included in this study. Most patients were male (105, 88%), with a median age of 22 years (18-25). 48.7% (58) of all patients had previously received pegylated interferon (PEG-IFN)-based therapy. Laboratory investigations, clinical parameters, and baseline findings are given in Table 1.

**Table 1:** Clinical characteristics and laboratory investigations of the study patients PEG-IFN: Pegylated interferon; HDV: Hepatitis D virus; HBV: Hepatitis B virus; HBeAg: Hepatitis B e-antigen.

Clinical parameters	n (%) or median (range)
Gender:male	105 (88%)
Age: (years)	22 (18-25)
Body mass index (kg/m^2^)	21.2 (15-33.9)
Time since hepatitis B diagnosed (months)	36 (<1-240)
Time since hepatitis D diagnosed (months)	13 (<1-168)
Previous PEG-IFN-based therapy Nonresponders Relapsers	58 (48.7%) 46 (38.6%) 12 (10.1%)
Investigations	
HDV-RNA positive	119 (100%)
HBV-DNA positive	83 (70%)
HBeAg negative	99 (83%)
HDV RNA quantitative: (log10 IU/ml) (n = 97)	5.67 (1.94-8.83)
HBV DNA quantitative: (log10 IU/ml) (n = 73)	3.12 (1.00-8.00)
Hemoglobin: (g/dl)	13.9 (7.4-16.7)
Total leucocyte count: (10^9^/L)	6.0 (2.4-15.4)
Platelets: (10^9^/L)	190 (26-443)
International normalization ratio:	1.01 (0.8-2.5)
Bilirubin: (mg/dL)	0.7 (0.2-10.5)
Alanine aminotransferase: (IU/mL)	69 (25-723)
Aspartate aminotransferase: (IU/mL)	54 (22-470)
Gamma glutamyl transferase: (IU/mL)	40 (10-942)
Alkaline phosphatase: (IU/mL)	
Albumin: (g/dL)	4.1 (1.8-4.8)
Creatinine (mg/dL)	0.8 (0.3-1.9)
Sodium (mmol/L)	139 (121-144)

HBV-DNA was detectable in 83 (70%). HBeAg was non-reactive in 99 (83%). This study only included chronic hepatitis D patients with detectable HDV RNA (qualitative). Out of these, 97 had a quantitative HDV RNA test as well. The median HDV RNA level in this latter group was 5.67 log10 IU/ml.

Forty-nine (41.2%) patients had an APRI score of more than one. Cirrhosis was identified in 45 of these patients. Among the cirrhotics, nine (7.5%) were categorized as Child B or Child C. Twenty-four (20.2%) had a Model For End-Stage Liver Disease (MELD) score of ≥10 and out of these 16 had a score of 15 or more. The anticipated clinical outcomes and risk of decompensation were evaluated using the Baseline-event-anticipation (BEA) score. Eight (6.7%) patients were categorized as BEA class A (mild risk of decompensation), 105 (88.2%) as BEA class B (moderate risk), and six (5.0%) as BEA class C (severe risk). The assessment of disease severity in this cohort is given in Table 2.

**Table 2:** Assessment of disease severity in young hepatitis D virus (HDV) infected patients Values are median (range) or number (percentage). MELD: Model For End-Stage Liver Disease

Characteristic	Number of Patients (%)
Splenomegaly	25 (21%)
AST to Platelet Ratio Index (APRI) APRI > 1.0	0.8 (0.2-12.2) 49 (41.2%)
Cirrhosis of liver	45 (37.8%)
Child class of cirrhosis (n = 45)	
A	36 (80%)
B	7 (15.6%)
C	2 (4.4%)
Decompensated disease	5 (4.2%)
Ascites	4 (3.4%)
Hepatic encephalopathy	1 (0.8%)
Median MELD score in cirrhotic patients	8 (6-27)
MELD ≥ 10	24 (20.2%)
MELD ≥ 15	16 (13.4%)
Baseline-event-anticipation (BEA) score (all patients = 119)	2 (1-6)
BEA-A	8 (6.7%)
BEA-B	105 (88.2%)
BEA-C	6 (5.0%)

A comparison of patients with and without cirrhosis was done. Key predictive parameters in patients with cirrhosis were splenomegaly, low total leucocyte count and low platelets, higher bilirubin, aspartate aminotransferase, gamma-glutamyl transferase and international normalization ratio (INR), low albumin, higher AST to platelet ratio index (APRI), and a higher BEA class (Table 3).

**Table 3:** Comparison of hepatitis D patients with and without cirrhosis Values are mean ± standard deviation or number (%) * Significant p-values by independent sample Mann-Whitney U test HDV: Hepatitis D virus; HBV: Hepatitis B virus; HBeAg: Hepatitis B e-antigen; INR: International normalization ratio.

Characteristic	Cirrhosis (n = 45)	Without cirrhosis (n = 74)	p-value
Male gender	40 (89%)	65 (88%)	1.000
Age	21.69 ± 2.72	21.47 ± 2.80	0.754
Duration of hepatitis B	64.13 ± 60.64	48.70 ± 52.86	0.167
Duration of hepatitis D	34.16 ± 44.09	23.38 ± 26.93	0.438
Body mass index	20.88 ± 3.30	21.80 ± 3.97	0.191
Splenomegaly	24 (53%)	1 (1.3%)	<0.001*
Spleen Size	13.46 ± 2.89	10.81 ± 0.95	0.001*
Previous treatment	23 (51%)	35 (47%)	0.686
Hemoglobin (g/dl)	13.36 ± 1.91	13.76 ± 1.55	0.375
Total leucocyte count (TLC): (109/L)	5.55 ± 2.31	7.04 ± 2.47	<0.001*
Platelets count: (109/L)	120.80 ± 62.98	227.73 ± 70.76	<0.001*
HDV RNA quantitative: (log10 IU/ml) (n = 97)	5.55 ± 1.57 (n = 37)	5.80 ± 1.56 (n = 60)	0.501
HBV DNA detected	27 (56%)	56 (76%)	0.071
HBV DNA quantitative: (log10 IU/ml) (n = 73)	2.96 ± 1.68 (n = 25)	3.48 ± 2.00 (n = 48)	0.309
HBeAg	7 (15%)	13 (18%)	0.776
Bilirubin (mg/dL)	1.48 ± 2.11	0.76 ± 0.45	0.038*
Alanine aminotransferase (ALT) (IU/L)	102.07 ± 99.54	105.80 ± 100.81	0.417
Aspartate aminotransferase (AST) (IU/L)	96.18 ± 92.95	73.69 ± 68.89	0.035*
Gamma glutamyl transferase (IU/L)	104.13 ± 159.10	56.27 ± 66.51	0.008*
Alkaline phosphatase (IU/L)	138.71 ± 68.40	132.41 ± 71.98	0.182
Albumin (g/dL)	3.77 ± 0.59	4.23 ± 0.33	<0.001*
INR	1.2538 ± 0.37	1.05 ± 0.13	0.005*
Creatinine (mg/dL)	0.85 ± 0.25	0.81 ± 0.15	0.650
Sodium (mmol/L)	136.71 ± 5.38	138.93 ± 2.20	0.098
AST to Platelet Ratio Index (APRI)	2.51 ± 2.22	0.88 ± 0.92	<0.001*
Baseline-event-anticipation (BEA) score			
BEA-A	0 (0%)	8 (11%)	0.001*
BEA-B	39 (87%)	66 (89%)	
BEA-C	6 (13%)	0 (0%)	

**Figure 1:**
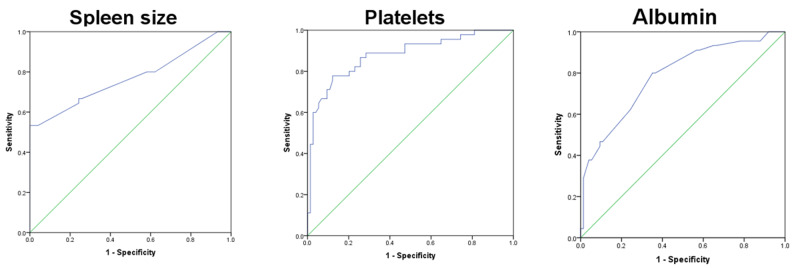
The association of spleen size (cm), platelet count, and albumin levels with cirrhosis. The areas under the ROC curves for these variables are 0.768, 0.878, 0.786, respectively. ROC: Receiver operating characteristic

Linear regression analysis by stepwise method revealed that splenic size (cm), platelet count, and albumin levels were independently associated with cirrhosis with p-values of <0.001, <0.001, and <0.003 and Beta coefficients of 0.391, -0.356, and -0.221, respectively. The areas under the ROC curves for these variables were 0.768, 0.878, and 0.786, respectively (Figure 1).

Binomial logistic regression with all three variables (splenic size, platelet count, and albumin levels) or with splenic size and platelet counts alone was also able to accurately predict cirrhosis. Both models were significantly better than the null model (Chi-Square Test, p < 0.05). The splenic size and platelet count contributed to most of the accuracy of the model. The equations used to calculate log-odds for cirrhosis were derived from this analysis and are given below for each model:

3 variable model

ln(P0c/1- P0c) = 0.842 *(Splenic Size in cm) - 1.482*(Albumin) - 0.023*(Platelet count) - 0.324

2 variable model

ln(P0c/1- P0c) = 0.828 *(Splenic Size in cm) - 0.027*(Platelet count) - 5.094

Where P0cis is the probability of cirrhosis and ln is the natural log.

Splenic size is in cm and platelet count in xyz x 109/L

**Figure 2:**
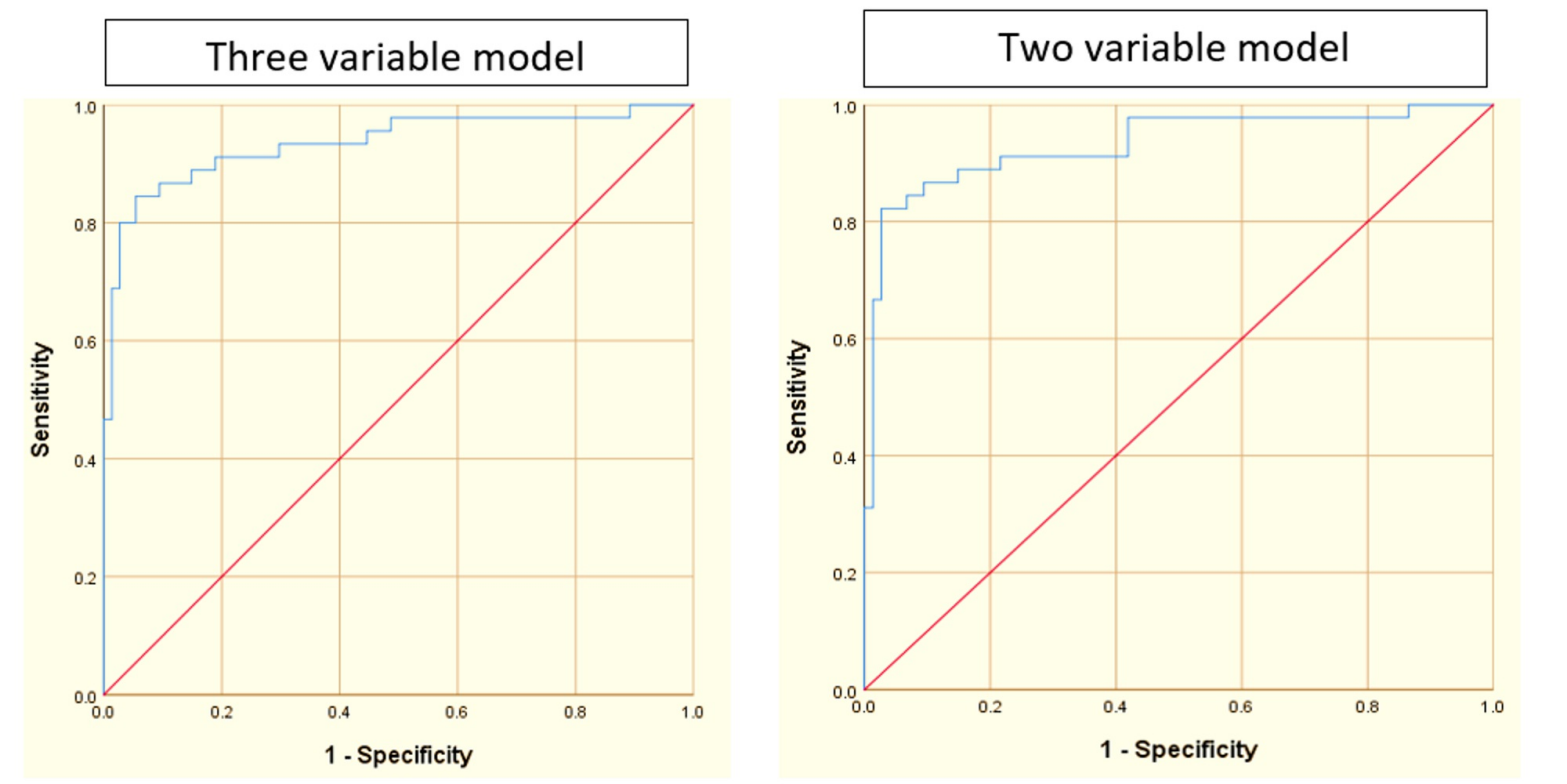
Results of binomial Logistic Regression. ROC curves for the three-variable and two-variable models. The areas under the ROC curves for these models are 0.935 and 0.932, respectively. ROC: Receiver operating characteristic

The right-hand side of the equation can be independently considered as a new scoring system. ROC curves have constructed using the results from each model (Figure 2).

The area under the receiver operating characteristics (AUROC) was 0.935 for the three-variable model and 0.932 for the two-variable model. The cut-off for the prediction of cirrhosis, for both models, can be taken as a value between -0.55 and +0.55 of the right-hand side of the equation, i.e., values greater than +0.55 highly predictive of cirrhosis and values less than -0.55 less likely to be cirrhosis. This has the highest combination of sensitivity and specificity (1.795).

Finally, patients who had previously received pegylated interferon therapy, displayed lower bilirubin (0.90 ± 0.97 vs 1.16 ± 1.68), lower alanine aminotransferase (ALT) 85.0 ± 54 vs 122.7 ± 12, lower aspartate aminotransferase (AST) (65.6 ± 40 vs 97.9 ± 101), and lower APRI (1.17 ± 1.16 vs 1.81 ± 2.10) when compared with patients without treatment, despite having a longer duration of disease (in months since HBV diagnosis: 72.14 ± 56.66 vs 37.80 ± 50.73 and HDV diagnosis 37.98 ± 36.63 vs 17.44 ± 29.64), (p < 0.05 each).

## Discussion

Hepatitis D, driven by genotype 1 HDV is a growing burden in Pakistan [12,13]. Various studies from across the country have shown a prevalence of 14-18% of HDV infection in HBsAg positive individuals [2,14,15]. However, the prevalence of anti-HDV antibodies is much higher; up to 37-38% in HBsAg positive patients with chronic liver disease, visiting liver clinics or hospitals for treatment, seem to harbour the HDV virus [16,17].

The presence of childhood HBV infection appears to be the main risk factor in this population. Chronic HBV infection makes young patients susceptible to the horizontal transmission of HDV in high prevalent areas. HBV/HDV mother-to-child co-transmission is rare [8,18]. These findings from Pakistan are broadly similar to those from a study conducted in a remote rural community in central Africa, where a high prevalence of HBsAg carriage and HDV infection in children and adolescents was observed [19]. This is in contrast to the observations from Romania, an Eastern European nation that still has high morbidity from HDV infection, but where the rate of infection has slowed and most patients are more than 50 years of age [20]. Hepatitis Delta International Network (HDIN) data, systematically analyzed by Wranke et al. confirms this disparate observation [21]. On average, hepatitis D patients from South Asia (Pakistan) were younger (32.7 years; range: 11-70) than those from Eastern Europe (35.7 years; range: 1-79). The HDIN data also showed that although HBeAg was negative in the vast majority of hepatitis D cases worldwide (10.3%-16.1% were positive), patients from Pakistan were more likely to be HBeAg positive (35.2%), and HBV DNA positive (60.8%).

Although it is accepted that the HDV causes a more severe infection at a younger age than HBV monoinfection, studies have not adequately tracked disease progression in the younger population. Indeed, while many studies done in Pakistan have shown that many hepatitis D patients are young adults (21-40 years) [15,22], none have studied the disease exclusively in the 18-25 year demographic.

We previously reported that children with HDV infection have a more aggressive form of liver disease than those with HBV monoinfection [10]. This was observed irrespective of HBeAg status. A comparison of this current study with the previous one done on children shows that male preponderance persists in young adults. However, we accept that this skew may arise from several cultural factors that bias attendance at our clinics. The comparative analysis also showed that the HBV DNA was detectable in about 50% of children and 70% of young adults. These patients had low HBV load (median 3.12 log10 IU/ml), which is expected, given the known suppressive effect of HDV on HBV [13]. On the other hand, HBeAg positivity decreased from 52% in children to 17% in the young adults' group. Cirrhosis was present in 37.8% of young adults versus 27% of children. It may be that there is an early aggressive phase in children, adolescents, and young adults followed by a more indolent progression. Moreover, there might be two categories of young adults, classified according to the aggressiveness of the disease. The ones with more aggressive disease progressed to cirrhosis at a very rapid pace.

The clinical outcomes associated with HDV infection can be predicted by the baseline-event-anticipation score (BEA score) [11]. The BEA score includes age, sex, the region of origin, bilirubin, platelets, and INR. The problems with employing this scoring system in our study are multifold. In our study cohort, all patients were from Pakistan, hence automatically gained a point. The cohort was below the age of 40 and so a point could not be added. The majority were also male, so they gained one additional point. All these factors limited the usefulness of the BEA score as a predictive tool. Indeed, as a result of these automatic point assignments, most patients fell into the category of moderate risk for poor long-term outcomes. Nonetheless, the scoring system and its classification of the majority is partially vindicated by the presence of cirrhosis in many patients and closely corresponds to the high APRI score in this cohort.

We also propose some parameters in this specific population that can help predict cirrhosis. Previously, the Delta-4 Fibrosis Score was proposed and validated for the accurate prediction of cirrhosis. This score utilized gamma-glutamyl transferase (GGT), ALT, platelet count, and liver stiffness and demonstrated an AUROC of 0.94 in a cohort of 77 HDV positive patients [23]. A similar score, called the Delta Fibrosis score, utilized GGT, age, albumin, and serum cholinesterase, and had an AUROC of 0.87 in a cohort of 100 HDV positive patients [24]. While these scoring systems are very informative, we found that a combination of spleen size and platelet combination is sufficiently sensitive and specific to classify cirrhotics in our demographic. The addition of albumin led to only partial gains in overall accuracy. Further testing may be required to validate this new model, but this new scoring system presents as a simplified additional tool, that utilizes non-invasive tests for the prediction of cirrhosis in young adults age 18-25 years with hepatitis D infection.

Finally, it is worth noting that 48.7% of our patients had received pegylated interferon therapy without sustained response. The lack of response is not surprising. Unlike the South American genotype 3 HDV, the Pakistani genotype 1 HDV does not respond well to pegylated interferon therapy [25-27]. However, in comparison to untreated patients, those who received pegylated interferon had lower bilirubin, transaminases, and a lower APRI. It may be that although sustained suppression of HDV is difficult, there is still some benefit of the treatment in slowing down the process of inflammation and fibrosis.

## Conclusions

In conclusion, our study of 119 hepatitis D patients uncovered key characteristics of this hitherto unstudied cohort. We show that most young adults with hepatitis D of age group 18-25 years in Pakistan are male. About two-thirds of them had HBV DNA detectable but most of them were HBeAg negative. Both factors did not influence the clinical outcome. High values of AST and ALT persisted in this demographic, indicating ongoing inflammatory activity. About one-third of patients had already developed cirrhosis and the rest of the patients were at moderate risk of disease progression. This paints a troubling picture in this demographic. These findings make it incumbent to address the risk factors associated with HDV infection and to implement an effective vaccination program for hepatitis B.
